# Toxicogenomics in non-mammalian species—Editorial

**DOI:** 10.3389/fgene.2012.00216

**Published:** 2012-10-16

**Authors:** Stephen Stürzenbaum, Michael Aschner, Jonathan H. Freedman

**Affiliations:** ^1^Analytical and Environmental Science Division, School of Biomedical Sciences, King's College LondonLondon, UK; ^2^Vanderbilt University Medical CenterNashville, TN, USA; ^3^Laboratory of Toxicology and Pharmacology, National Institute of Environmental Health Science, National Institute of Health, Research Triangle ParkNC, USA

In October 2004, the Organisation for Economic Co-operation and Development (OECD) and the International Programme on Chemical Safety (IPCS) organized a workshop in Kyoto Japan to outline the fundamental aspects and future directions of the then emerging science of “Toxicogenomics.” The output of this three-day meeting was summarized in the Report of the OECD/IPCS workshop on Toxicogenomics (OECD Series on Testing and Assessment, Number 50, http://www.who.int/ipcs/methods/oecd_report.pdf). In essence, Toxicogenomics was defined as any study that investigates the response of a genome to hazardous substances by means of (1) genomic-scale mRNA expression analyses (Transcriptomics), (2) cell and tissue wide protein expression techniques (Proteomics), or (3) cell and tissue wide metabolite profiling (Metabolomics). These “omic” datasets typically require intricate *in silico* analyses (Bioinformatics) to integrate the results and by doing so provide insights into mechanistic toxicology and biomarkers of exposure.

Since 2004, the initiation, and indeed completion, of numerous whole genome-sequencing projects has driven the exponential growth of available genetic information. Collating this vast amount of data into functional and mechanistically meaningful units is providing novel insights into pathogenesis, new methods of risk assessment, genetic risk-modifications in preventative medicine, and new therapeutic targets for pharmaceutical and biological medicines. Most toxicogenomic responses, however, are multi-dimensional due to the facts that toxicants usual affect multiple intra-and extra-cellular targets and occur as complex mixtures, which are inherently difficult to decipher. Some molecules or conditions are exclusively toxic to biological systems and classified as being non-essential, while others are essential for life. According to Paracelsus, above certain threshold even the essential will become toxic. Thus, tightly controlled homeostatic control mechanisms are vital drivers of well-being, longevity, and survival. The identification and characterization of these mechanisms and the cognate regulatory pathways form the foundations of Toxicogenomics.

This Research Topic issue explores our current knowledge pertaining to the multitude of genomic and toxicological tools within non-mammalian organisms, arguably an underdeveloped niche. This e-book begins with five seminal reviews on (1) the yeast system, which focusses on genome-wide responses to chemical stressors linked to Environmental Health, Pharmacology, and Biotechnology (dos Santos et al., 2012); (2) the fruit fly *Drosophila melanogaster*, as a model for lead neurotoxicology and Toxicogenomics (Hirsch et al., 2012); (3) the nematode *Caenorhabditis elegans*, to investigate the genome-wide response to metal exposure (Caito et al., 2012); (4) next generation sequencing approaches in fish Toxicogenomics (Mehinto et al., 2012); and (5) the application of Toxicogenomics in amphibians (Helbing, 2012). These reviews set the stage for five research articles that describe how genomic tools have aided in uncovering target mechanisms of (eco)toxicological importance. These comprise transcript meta-analyses in *C. elegans* to pinpoint humic acid, quercetin, and tannic acid-mediated effectors of stress and aging (Pietsch et al., 2012 and Menzel et al., 2012); the assessment of a municipal landfill soil via a functional environmental genomic approach using the springtail *Folsomia candida* (Roelofs et al., 2012); a bioinformatic approach to model genotoxic chemical mutations in *Drosophila melanogaster* (Cingolani et al., 2012); and a study that investigates the influence of nitrate and nitrite on targeted gene expression in the tadpole *Rana catesbeiana* (Hinther et al., 2012).

By spanning a wide taxonomic breadth, this encyclopaedic coverage results in a collection of unique approaches, tools and technologies, which are currently defined by the availability and feasibility for each organism to study the genomics of xenobiotic or stress biology. We anticipate that this information will not only foster cross-phyla awareness, but also expand the horizon of Toxicogenomics. We hope you enjoy this eclectic mix of papers.

## References

[B1] Caito S. Fretham S. Martinez-Finley E. Chakraborty S. Avila D. Chen P. (2012). Genome-wide analyses of metal responsive genes in *Caenorhabditis elegans*. Front. Gene. 3:52 10.3389/fgene.2012.00052 22514555 PMC3322339

[B2] Cingolani P. Patel V. M. Coon M. Nguyen T. Land S. J. Ruden D. M. (2012). Using *Drosophila melanogaster* as a model for genotoxic chemical mutational studies with a new program, SnpSift. Front. Gene. 3:35 10.3389/fgene.2012.00035 22435069 PMC3304048

[B3] dos Santos S. C. Teixeira M. C. Cabrito T. R. Sá-Correia I. (2012). Yeast toxicogenomics: genome-wide responses to chemical stresses with impact in environmental health, pharmacology, and biotechnology. Front. Gene. 3:63 10.3389/fgene.2012.00063 22529852 PMC3329712

[B4] Helbing C. C. (2012). The metamorphosis of amphibian toxicogenomics. Front. Gene. 3:37 10.3389/fgene.2012.00037 22435070 PMC3303113

[B5] Hinther A. Edwards T. M. Guillette L. J. Jr. Helbing C. C. (2012). Influence of nitrate and nitrite on thyroid hormone responsive and stress-associated gene expression in cultured *Rana catesbeiana* tadpole tail fin tissue. Front. Gene. 3:51 10.3389/fgene.2012.00051 22493607 PMC3318185

[B6] Hirsch H. V. B. Lnenicka G. Possidente D. Possidente B. Garfinkel M. D. Wang L. (2012). *Drosophila melanogaster* as a model for lead neurotoxicology and toxicogenomics research. Front. Gene. 3:68 10.3389/fgene.2012.00068 22586431 PMC3343274

[B7] Mehinto A. C. Martyniuk C. J. Spade D. J. Denslow N. D. (2012). Applications of next-generation sequencing in fish ecotoxicogenomics. Front. Gene. 3:62 10.3389/fgene.2012.00062 22539934 PMC3336092

[B8] Menzel R. Menzel S. Swain S. C. Pietsch K. Tiedt S. Witczak J. (2012). The nematode *Caenorhabditis elegans*, stress and aging: identifying the complex interplay of genetic pathways following the treatment with humic substances. Front. Gene. 3:50 10.3389/fgene.2012.00050 22529848 PMC3328794

[B9] Pietsch K. Saul N. Swain S. C. Menzel R. Steinberg C. E. W. Stürzenbaum S. R. (2012). Meta-analysis of global transcriptomics suggests that conserved genetic pathways are responsible for Quercetin and Tannic acid mediated longevity in *C. elegans*. Front. Gene. 3:48 10.3389/fgene.2012.00048 22493606 PMC3319906

[B10] Roelofs D. de Boer M. Agamennone V. Bouchier P. Legler J. van Straalen N. (2012). Functional environmental genomics of a municipal landfill soil. Front. Gene. 3:85 10.3389/fgene.2012.00085 22623925 PMC3353140

